# Multiple Genetic Associations with Irish Wolfhound Dilated Cardiomyopathy

**DOI:** 10.1155/2016/6374082

**Published:** 2016-12-13

**Authors:** Siobhan Simpson, Mark D. Dunning, Serena Brownlie, Janika Patel, Megan Godden, Malcolm Cobb, Nigel P. Mongan, Catrin S. Rutland

**Affiliations:** ^1^Faculty of Medicine and Health Sciences, School of Veterinary Medicine and Science, The University of Nottingham, Sutton Bonington Campus, Loughborough LE12 5RD, UK; ^2^Department of Pharmacology, Weill Cornell Medical College, 1300 York Avenue, New York, NY 10065, USA

## Abstract

Cardiac disease is a leading cause of morbidity and mortality in dogs and humans, with dilated cardiomyopathy being a large contributor to this. The Irish Wolfhound (IWH) is one of the most commonly affected breeds and one of the few breeds with genetic loci associated with the disease. Mutations in more than 50 genes are associated with human dilated cardiomyopathy (DCM), yet very few are also associated with canine DCM. Furthermore, none of the identified canine loci explain many cases of the disease and previous work has indicated that genotypes at multiple loci may act together to influence disease development. In this study, loci previously associated with DCM in IWH were tested for associations in a new cohort both individually and in combination. We have identified loci significantly associated with the disease individually, but no genotypes individually or in pairs conferred a significantly greater risk of developing DCM than the population risk. However combining three loci together did result in the identification of a genotype which conferred a greater risk of disease than the overall population risk. This study suggests multiple rather than individual genetic factors, cooperating to influence DCM risk in IWH.

## 1. Introduction

Dilated cardiomyopathy (DCM) is the most common cause of cardiac death in Irish Wolfhounds (IWH) [1, 2]. In human DCM there is often a heritable genetic basis of this disease [3, 4]. In several dog breeds, including IWH, canine DCM has been shown to have heritable component [5–7]. The heritability of canine DCM and its clinical resemblance to human DCM suggest that there is also a genetic basis to canine DCM [8, 9]. The IWH is a rare, giant breed of dog and it is considered to be vulnerable by the UK kennel club due to the small number of annual puppy registrations [10]. IWHs are not long lived with a median age at death in the UK population reported to be 7.04 years and a maximum reported age of 11.83 years [11]. In the same survey of 165 other breeds, only 14 had a lower median age at death than the IWHs and most were large or giant breeds. There were 71 breeds with a median age at death higher than the IWH maximum reported age at death [11].

There are a number of health problems reported to occur frequently in the IWH breed and may contribute to the low median age at death. These include heart disease, osteosarcoma, bronchopneumonia, and gastric dilation-volvulus [1, 12–14]. Canine osteosarcoma often results in euthanasia due to poor quality of life in affected dogs and survival following diagnosis is typically less than one year [12, 15, 16]. In contrast, the prognosis of dogs with heart disease such as atrial fibrillation or DCM is better when diagnosed and treated early with median time to death reported to be up to 4 years following early heart disease diagnosis [17]. There is limited literature on pneumonia in IWH [18, 19] but Greenwell and Brain [13] reported a breed predisposition compared to other breeds treated for pneumonia within a specialist small animal hospital. The majority (88.9%) of these initial cases were successfully treated, although 44% were ultimately euthanized due to recurrent pneumonia [13]. Although treatable, gastric dilation-volvulus is often life threatening, with reported mortality rates between 20.5% and 30% across all breeds [20, 21]. Osteosarcoma and heart disease are the most likely causes of death or euthanasia but increased incidence of pneumonia and gastric dilation-volvulus relative to other breeds also lower the median lifespan of IWH [1, 12–14]. Cardiac disease is the most common specific reported cause of death in IWH, thus improving the prevention and diagnosis, and treatment of cardiac disease is likely to have the greatest impact on improving longevity and welfare in the breed [22].

There have been several studies investigating the genetic basis of canine DCM but despite this, very few genetic associations have been identified [8, 23–26]. Possible reasons for the lack of identified genetic associations include the use of inappropriate control populations, incomplete clinical characterisation of control and affected populations, inadequate samples sizes, or an assumption of simple- Mendelian inheritance, despite the evidence suggesting more complex multifactorial influences on heritability [5]. Inappropriate controls have included unaffected individuals from different breeds to the affected individuals, or young individuals that still have the potential to develop disease [27–29]. Many canine DCM studies have also been limited by small sample sizes of between 5 and 40 individuals, which is unlikely to be sufficient to detect genetic associations [30]. Furthermore, our previous studies have shown that multiple loci likely cooperate to influence canine DCM development; thus combinations of genetic factors should be examined together as well as individually for associations with disease [31]. It is also important to note that different breeds can have differing aetiologies and thus may also have differing genetic associations or causes. This current study utilises the largest recorded number of affected IWH combined with appropriately aged unaffected breed-matched controls and examines multiple loci for an association with canine DCM together as well as individually.

In addition to commonly being diagnosed with DCM, IWHs are frequently diagnosed with atrial fibrillation (AF) [2, 32]. Despite the presence of AF in a large percentage of dogs with DCM, the mechanistic and clinical relationship between DCM and AF has not been clarified [2, 32–34]. Although AF is not associated with the development of DCM in people, the presence of AF in people with DCM and heart failure has been shown to negatively influence survival [35, 36]. IWH can develop DCM without AF, though it seems that <2% of IWHs with AF do not go on to develop DCM [2, 32]. If AF is a potential precursor to DCM, the time from diagnosis of AF to DCM is important; if it is several years then the presence of AF could be less of a concern than if it is merely a few months. Also if AF is a precursor to DCM there is the potential to give individuals diagnosed with AF drugs such as the phosphodiesterase III/calcium sensitizing drug, pimobendan, to improve survival [17]. In addition to the potential clinical implications of AF diagnosis and potential early interventions for both dogs and humans, if AF can be shown to be related to DCM for genetic association testing, this diagnosis can be used to test for associated DCM genetic implication. This also has implications for individuals included in the unaffected group for genetic association testing; individuals included in the unaffected group must be free of both DCM and AF.

There is evidence that DCM affects males more often and/or earlier in life than females [32, 34, 37, 38]. Previous IWH DCM genetic association studies have either not reported the age of their unaffected IWHs or used arbitrary ages to define the unaffected control group [23, 28, 39]. Brownlie and Cobb [32] found a difference between the sexes in the age of onset of DCM in IWH in 39 individuals. If the age of onset differs between males and females, the age at which an unaffected individual can be included in the unaffected group for genetic association studies should also be different for males and females. If DCM affects males more often than females or there are differences in the age of onset this raises important questions as to why there might be a difference between the sexes in age or frequency of onset. This study therefore also aims to confirm whether there is a sex effect and/or age of onset differential in relation to DCM in the IWH.

In this study, we statistically tested the relationships between DCM and AF, sex differences in disease prevalence and age of onset, and the use of multiple genetic loci to establish genetic associations in IWHs. The hypothesis that AF is associated with DCM in IWHs was tested by establishing how many individuals diagnosed with DCM were also diagnosed with AF and how long an individual can have a diagnosis of AF prior to being diagnosed with DCM. Individuals diagnosed with AF, but not DCM, were also tested to establish whether they had AF diagnosed for a longer period of time on average than individuals which subsequently developed DCM. The second hypothesis was that male IWHs were diagnosed with both DCM and AF more frequently than females and secondary to that, the combined incidence of both disorders was higher in males than females. In addition, the hypothesis that the age of onset of DCM and AF was different in males as compared to females was tested. Therefore, the age at which an individual can be considered unaffected was established for males and females. The fourth hypothesis tested was whether multiple loci could act together to cause DCM; thus examining loci individually may not yield significant results [31]. In cases where a locus is associated with DCM, the result may not have clinical implications; for example, a locus may be associated with disease, but only explaining a small number of cases. However, the examination of multiple loci in combination could improve the reliability and clinical value of genetic associations. The hypothesis that genotypes at multiple loci more comprehensively predict incidences of DCM and AF than individual loci alone was tested by genotyping multiple loci putatively associated with IWH DCM and testing for associations at loci both individually and in combination.

## 2. Methods

### 2.1. Samples and Health Updates

This study was approved by the University of Nottingham ethics committee ethical number 1823 160714 in compliance with the Home Office regulations and the Veterinary Surgeons Act. Informed consent was obtained from all dog owners involved in this research. Buccal swabs from 379 IWHs were taken using Isohelix DNA Buccal Swabs (Cell Projects Ltd. UK). The swab was inserted into the dog's mouth by either the owner, vets, or trained members of the research group and gently rubbed on the cheek for up to 2 minutes to enable optimum cell collection. Swabs were stored as directed by the manufacturer, at room temperature upon collection followed by 4°C. Any swabs stored for longer than a month prior to DNA extraction were stored at −20°C. Optimisation trials indicated that appropriate quality and quantity of DNA could be obtained following all storage methods. When the buccal swabs were taken owners completed a short information sheet for each dog in order to obtain current and past health information, date of birth, pedigree information, sex, and neutering status. All information was associated with a unique sample number and stored in a secure excel database.

Follow-up data on the health of DNA-sampled dogs was obtained at numerous time points. Owners were asked to fill in a “health update” form annually or following any change in health/upon death. Forms could be completed either online or in paper format to ensure maximum owner participation. In addition, telephone surveys were carried out on this longitudinal health study. The surveys were utilised to obtain health updates and epidemiology data on all dogs from which samples had been obtained. Many IWH are presented to veterinary cardiologists on a regular basis for heart disease screening. These heart screenings consist of cardiac auscultation, a six-lead electrocardiogram, and complete echocardiographic assessment. Data from these heart screenings were provided by veterinary cardiology specialist Dr. Serena Brownlie. Cardiac diagnoses were determined by Professor Malcolm Cobb based on the data from heart screenings. Information from owner health updates and heart screenings was analysed alongside genetic data.

### 2.2. DCM and AF Relationship

**Figure 1:**
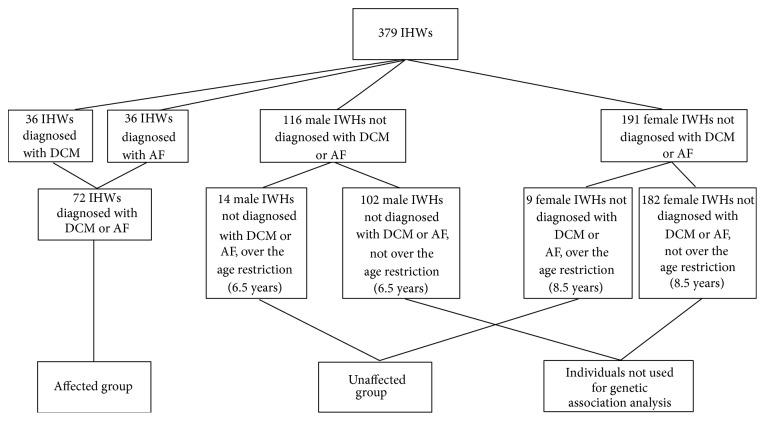
Study cohort.

Individuals diagnosed with DCM, AF, and both DCM and AF in the database were identified, and the numbers in each diagnosis category “DCM,” “AF,” and “DCM and AF” were determined (Figure 1). In the subset of individuals with both DCM and AF, the time from AF diagnosis to DCM diagnosis was established based on the dates of the heart screenings at which each was diagnosed. Individuals that were diagnosed with AF, but not DCM, had the time with AF determined by the time between the first heart screening that AF was diagnosed at and the most recent heart screening. A *t*-test was performed to establish if the time from AF diagnosis to DCM diagnosis was significantly different from the time that individuals had been known to have a diagnosis of AF, but not DCM.

### 2.3. Sex Differences in Numbers Affected and Age of Disease Onset

The number of males and females diagnosed with DCM or AF was established from the database along with the number of males and females not diagnosed with DCM or AF. A *χ* ^2^ test was performed to establish whether there were differences in the number of males and females diagnosed with DCM or AF. The age at first diagnosis of DCM or AF was established for all individuals diagnosed with either disorder. A *t*-test was performed to identify statistical differences between males and females at age of diagnosis. The data was also displayed visually in bar plots generated using R statistical software. In addition, a *χ* ^2^ test was performed to establish whether there were differences in the number of males and females affected which had been neutered or not neutered.

### 2.4. Genetic Associations

#### 2.4.1. DNA Extraction and Restriction Fragment Genotyping

To extract DNA from buccal swabs, the end of the swab was first cut into small pieces using scissors which were sterilised between swabs using Anistel (Tristel, UK) and then wiped down with Industrial Methylated Spirits (IMS; Thermo Fisher Scientific, USA). The small pieces of swab were put into a sterile 1.5 mL microcentrifuge tube (Eppendorf, Germany). The DNA-containing swab fragments were resuspended in 600 *μ*L of a solution composed of 5% sodium Chelex® 50–100 mesh (dry; Sigma-Aldrich, USA) reconstituted in 1x TE (10 mM Tris (Thermo Fisher Scientific, USA), 1 mM EDTA (Thermo Fisher Scientific, USA) in H_2_O, pH 8.0) and 20 *μ*L of 10 mg/mL proteinase K (Promega, USA), 50 mM Tris (Thermo Fisher Scientific, USA), and 10 mM CaCl_2_ (Thermo Fisher Scientific, USA), pH 8.0. The sample-containing microcentrifuge tube was vortexed to mix the components. The microcentrifuge tube was then placed in a water bath at 45°C overnight to digest the cells. The sample was then vortexed once more before incubation in a heating block at 100°C for 8 minutes to inactivate the proteinase K. The sample was then centrifuged at 13,500 ×g for 3 minutes to separate the Chelex and swab from the supernatant. The DNA-containing supernatant was then transferred into a sterile 1.5 mL microcentrifuge tube and was used in subsequent PCRs. The extracted DNA was then stored at −20°C in the Nottingham Comparative Genomic Biobank.

Restriction digests were used to genotype SNPs previously shown to be associated with DCM [23], in the Nottingham IWH cohort. In the first instance the effect of the SNPs on restriction sites within the affected loci was examined. PCR primers were designed to flank five DCM-associated SNPs which alter restriction sites. Where the SNP did not alter or create a restriction site, PCR primers were designed to introduce a restriction site into the PCR product to detect the presence/absence of the SNP. Primer information and fragment size amplified are presented in Table 1. Primers were obtained from Sigma-Aldrich, UK. The SNPs are hereby referred to as Chr1 for rs21953123, Chr10 for rs22078677, Chr15 for rs22422063, Chr21 for rs22923291, and Chr37 for rs24025150.

The PCR reaction mixture consisted of the following: 1x LightCycler® 480 Probes Master Mix (Roche, Switzerland), primers at a concentration of 0.5 *μ*M, and between 2 and 4 *μ*L of template DNA per reaction, adjusted to 25 *μ*L using PCR grade H_2_O. Where PCR reactions initially failed, increased template DNA was used (up to 4 *μ*L) and the amount of H_2_O in the reaction was reduced accordingly. PCR reactions were carried under the following conditions: Initial denaturation 94°C for 30 seconds, 40 cycles of 94°C for 30 seconds, annealing temperature for 30 seconds, 72°C for 30 seconds, and final extension 72°C for 5 minutes. The annealing temperature optimisation was determined by performing a temperature gradient PCR between 52°C and 64°C (see Table 2).

Restriction digests were conducted on each PCR product. The reaction mixture for each restriction digest consisted of 3 *μ*L of 10x buffer, 20 *μ*L PCR product, and 1–6 units of enzyme in a reaction volume of 30 *μ*L. The enzyme manufacturer provided the appropriate buffer for each enzyme and the stated activation temperature; the number of units of enzyme was optimised for restriction digest reaction. The details for each restriction digest are shown in Table 3. The restriction digests were performed by incubating the reaction mixture at the activation temperature of enzyme for 14 hours.

Following digestion, the digested PCR products were electrophoresed at 100 v for 45 mins on 2% agarose 1x TAE gels stained with Nancy-520 (Sigma-Aldrich, USA) in 1x TAE buffer. A DNA size ladder (*ϕ*x-HaeIII fragments) purchased from NEB was used to determine the size of the digested PCR fragments. Genotypes were determined by the restriction digestion fragment pattern based on the presence of an appropriate restriction site determined by the presence of the SNP, with the possibility of detecting homozygotes (AA, aa) and heterozygotes (Aa). Presumptive homozygotes for each genotype (AA, aa) were confirmed by direct Sanger sequencing of representative PCR fragments (Source BioSciences, Nottingham). For all loci a single band, the same size as the PCR product, showed that none of the PCR product had been digested and was genotyped as a homozygote for the allele that does not digest. The absence of this larger band and the presence of smaller bands at the expected sizes following digestion (Table 1) showed that all of the PCR product had been digested so these were genotyped as homozygotes for the allele that was digested by the restriction enzyme. Those with a band the same size as the PCR product plus bands at the expected sizes following successful digestion in Table 1 were genotyped as heterozygotes. These genotypes were manually recorded within a database alongside appropriate sample numbers and clinical information. One sample of each genotype at each SNP was confirmed by Sanger sequencing by Source Bioscience (Nottingham, UK).

#### 2.4.2. Analysis

To establish whether the population within this study genetically resembled the published data by Philipp et al. [23], the allele frequencies at each locus were determined in the entire sampled population irrespective of age or disease status. These allele frequencies were compared to the published allele frequencies at the same loci.

**Figure 2:**
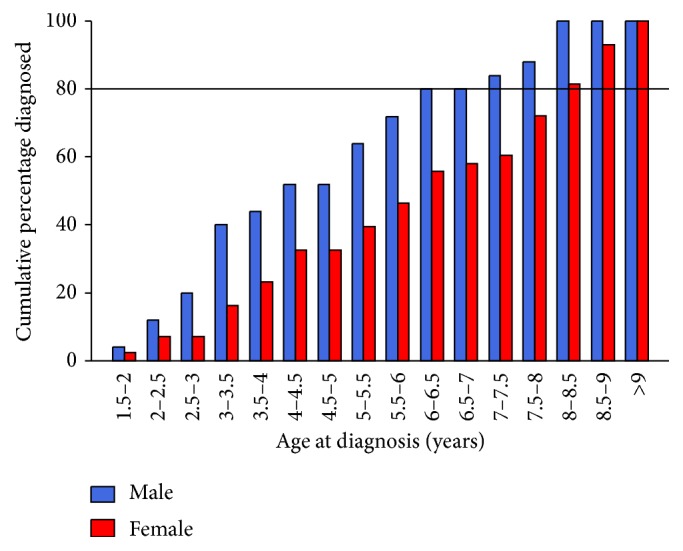
Cumulative percentage of individuals diagnosed with DCM or AF at or before each age category. Males (blue) and females (red) are shown separately.

All individuals with a diagnosis of DCM or AF were included in the affected group. As outlined in the results and Figure 2, males were shown to be affected by DCM and AF at an age earlier than females. For this reason, females were considered to be unaffected if they had not been diagnosed with either DCM or AF at a heart test after the age of 8.5 years. Males were considered unaffected if they had reached the age of 6.5 years and had a heart test without a diagnosis of DCM or AF. All affected animals were included regardless of age.

The number of individuals expected to develop DCM under four common penetrance models for each SNP was obtained by methods described by Camp [40]. The observed risk of disease for each genotype was obtained directly from the data by dividing the number of each genotype affected by the total number of individuals with the genotype. The genetic penetrance parameter (*γ*) was then established for each SNP. For the multiplicative, additive, and dominant models, *γ* indicates the relative risk of the heterozygote genotype compared to the homozygote genotype with the lowest risk; for the recessive model it indicates the relative risk of the most at risk homozygote compared to the least at risk homozygote. The penetrance parameter was then used to calculate the proportion of each genotype expected to develop disease under each model.  Table 4 shows how the penetrance parameter was used with the lowest homozygote risk under each model to calculate these proportions.

The proportion of each genotype expected to develop disease under each model was then translated into the number expected by multiplying the proportion by the total number of individuals observed with the genotype, irrespective of disease status. A *χ* ^2^-squared test was performed to establish which model of penetrance most closely fitted the observed data. The model with the smallest *χ* ^2^-squared value was taken to be the best fit of the observed data.

Having established the model of penetrance, the most appropriate test for association with disease was then carried out. Where A is the disease associated allele, for dominant models the counts for individuals which genotyped as a/A and A/A can be pooled as the penetrance is equivalent; similarly for recessive models the counts for individuals genotyped as a/a and a/A can be pooled [41]. For multiplicative models allelic tests of association are more powerful [41]. Additive models are most appropriately tested by a Cochran-Armitage trend test, or genotype association testing [41]. To test for association with disease *χ* ^2^ tests were performed. The expected numbers of affected individuals with each genotype or allele were obtained by establishing the proportion of unaffected individuals with each genotype or allele. These proportions of unaffected individuals with each genotype or allele were then multiplied by the total number of affected individuals to establish the expected number of affected individuals with each genotype. To account for multiple tests a Bonferroni correction was applied to the *p* values.

To establish whether particular genotypes gave an increased risk compared to the overall population risk, relative risks were calculated alongside 95% confidence intervals. Relative risks were calculated by dividing the genotype risk with the population risk and 95% confidence intervals were calculated using the methods in [42]. Where appropriate, genotypes were combined, for example, where the penetrance model was shown to be recessive or dominant, or where there were less than 5 total individuals with a genotype.

To establish whether using multiple loci to test for an association could increase the association compared to individual loci alone, each significant locus was considered with each of the other significant loci in pairs and all combined. For this analysis the expected numbers were calculated and tested for an association in the same way as for loci individually. Risks of disease for each genotype combination were also established and relative risks calculated in the same way as individual loci.

## 3. Results

### 3.1. DCM and AF Relationship

There were 36 individual dogs diagnosed with DCM included in the current study (from a total of 379 genotyped individuals, 9.50%, Figure 1) and 29 (80.5%) DCM affected individuals also had a diagnosis of AF. In total, 17 of the 29 (58.6%) were diagnosed with DCM and AF at the same time, whereas the remaining 12 had been diagnosed with AF prior to the DCM diagnosis. Of the 12 that had been diagnosed with AF prior to DCM, 11 (91.7%) had been diagnosed with AF within 2 years, and all had DCM diagnosed within 3 years of their first AF diagnosis.

In the current study, there were additional 36 individuals (from a total of 379 genotyped individuals, 9.50%) that had been diagnosed with AF, but not DCM. Of these individuals 24 (66.7%) had not been presented for heart disease screening since diagnosis. Only 7 (19.4%) had only been tested within 1 year of their original diagnosis of AF. Further 4 individuals (10.8%) had been tested within 2 years of original diagnosis and one individual had been tested 2.5 years following AF diagnosis. In total, heart disease screening data and DNA samples were taken across a ten-year period.

The time from AF diagnosis to DCM diagnosis in the group of individuals diagnosed with both AF and DCM was not significantly different from the time individuals only diagnosed with AF had been known to have a diagnosis of AF (*t* = 0.56, *p* = 0.58).

### 3.2. Sex Differences in Numbers Affected and Age of Disease Onset

It was noted that there was no difference in observed and expected numbers of affected cases in either males or females (*n* = 191 entire females and 66 neutered females, with 28 and 16 affected, resp. *n* = 116 entire males and 25 neutered males with 16 and 7 affected resp. *t* = 2.63 for females and 2.54 for males, *p* > 0.05).

Individuals with diagnoses of DCM and AF were included in the affected group. There was no difference in the proportion of males and females affected by DCM/AF combined, *χ* ^2^-squared test result = 0.13 (*p* = 0.72, df = 1). There was a significant difference between males and females in the age of diagnosis of DCM/AF combined. DCM/AF combined mean age at diagnosis was 4.82 years for males and 6.14 years for females (*t* = 2.43, *p* = 0.019). Of males that developed AF or DCM, 80% had done so before the age of 6.5 years, while 80% females that developed AF or DCM did so before the age of 8.5 (Figure 2). Using these age restrictions in the sample cohort there were 9 females unaffected by either diagnosis over the age of 8.5 years and 14 males unaffected over 6.5 years. Of the individuals not included in the DCM/AF affected group or the unaffected group, 20 were known to have died of causes other than DCM or AF before reaching the age restrictions, 234 had not reached the age restrictions, and 27 were over the age restriction but had not been heart tested since reaching the age restriction, and 3 were of unknown age. Using the information above, it was therefore possible to define the “unaffected” group with a high level of stringency and specificity, in addition to enhancing the knowledge on sex related differences in age of clinical diagnosis.

Within the study cohort (Figure 1), 23 (34.7%) individuals were diagnosed with DCM or AF at their first heart test and of these, 19 (82.6%) were less than 6.5 years old. Of the 4 that were over 6.5 years old at first diagnosis without a previous heart test 1 was male and 3 were female. Due to the uncertainty in the age that these older individuals would have developed the condition, these individuals were removed from the age of onset analysis to ensure that these do not skew the difference in age of onset between the sexes. The *t*-test result excluding these individuals remained significant at the 5% threshold level (*t* = 2.11, *p* = 0.040), which was comparable to when those individuals were included within the dataset.

### 3.3. Genetic Associations

The individuals in the current study were primarily from the UK IWH population, while those in the study by Philipp et al. [23] were from mainland Europe. Despite the use of a different subpopulation of IWHs there was a general concordance between the published allele frequencies and those in the current study (Table 5). The alleles at the SNPs on chromosomes 1, 10, and 37 were within 0.1 of the published frequencies. The alleles at the SNPs on chromosomes 15 and 21 were close to within 0.1 of the published frequencies.

The mode of penetrance was established to be multigenic for the SNPs on chromosomes 1 and 10: dominant for the chromosome 15 and 21 SNPs and recessive for the SNP on chromosome 37 (Table 6). This allowed allelic association tests to be performed for the SNPs on chromosomes 1 and 10, the numbers of individuals genotyped as AA and AC for SNP on chromosome 15 to be combined, and the numbers of individuals genotyped as GA and GG for the SNPs on chromosomes 21 and 37 to be combined; thus the number of degrees of freedom for each association test was reduced to one (Table 6).

Three loci had significant associations with DCM/AF combined, specifically the SNPs on chromosomes 1, 21, and 37 (Table 6). The risks of developing DCM or AF for each genotype are presented in Table 7, along with the total numbers of individuals with each genotype. There were very few individuals genotyped as TT at the chromosome 1 locus, CC was the highest risk allele, and allelic association tests established that the C allele was associated with DCM/AF; therefore the numbers of TT individuals were combined with the CT individuals. Chromosome 21 and 37 SNPs were determined to be dominant and recessive, respectively, so the GA and GG genotypes were combined at both loci. The overall risk of developing DCM or AF was 0.76. Several genotypes gave an elevated risk compared to the overall risk, and several gave reduced risk, none of these increased or decreased risks were significantly different from the overall risk.

Combining genotypes at pairs of loci decreased the significance of the chromosome 1 SNP but increased the significance of chromosomes 21 and 37 SNPs (Tables 6 and 8). Combining all three loci together increased the significance for all loci (*χ* ^2^-squared value = 41.09, *p* = 0.00000078).

From both the pairs of loci and all three loci combined, the genotypes with the greatest disease risk were those which combined the high risk individual genotypes (Tables 8 [Table tab9] [Table tab10] [Table tab11]–12). In particular Chr1 CC, Chr21 GA/AA, and Chr37 AA combined gave the single highest risk of developing disease and was significantly higher than the overall population risk. The opposite was also the case with the lowest combined disease risk resulting from the combined genotypes of those with individually the lowest risk, but none gave a significantly decreased risk compared to the overall population risk. All other genotype combinations are somewhere in between.

## 4. Discussion

The data acquired here supports an association between AF and DCM in IWHs. The majority (80.5%) of individuals with DCM also had a diagnosis of AF. Most individuals were diagnosed with AF at the same time as DCM, or in the 2 years prior to the diagnosis of DCM, which indicates that AF may be a precursor to a clinical diagnosis of DCM. The current results are consistent with a previous study which found that 87.6% of individuals diagnosed with DCM were also diagnosed with AF [2]. Further evidence for the relationship between AF and DCM comes from the time period that individuals diagnosed only with AF have spent with the AF diagnosis. The majority (66.7%) had not had a subsequent heart test and of those that had been retested the longest time between initial AF diagnosis and most recent heart test was 2.5 years. All individuals with AF alone still had/have the potential to develop DCM as the time periods with AF were not longer than the time periods between AF diagnosis and DCM diagnosis (*t* = 0.56, *p* = 0.58). This finding is consistent with Vollmar [2] in which 11 individuals were diagnosed with AF, but not DCM; however only 3 individuals were known not to have subsequently developed DCM, with 29 months as the maximum time with AF alone. This relationship between AF and DCM allowed for the inclusion of individuals diagnosed with AF as well as DCM in the affected group for genetic association tests. In addition, this provides evidence that individuals diagnosed with AF should be carefully monitored and regularly presented for heart testing to ensure that they do not yet require treatment for DCM. There is also the potential to improve the survival of individuals diagnosed with AF by treating them with drugs such as pimobendan prior to the development of DCM or heart failure [17], in addition to receiving therapy for AF.

Very few IWHs were unaffected by AF or DCM into old age (defined as older than the median lifespan of 7.04 years in IWHs [11]), as most individuals developed one or both disorders, or they died of other conditions before reaching old age. As noted earlier, this breed is also susceptible to osteosarcoma, pneumonia, and gastric dilation-volvulus, all of which can require euthanasia [12–14]. This influenced the number of animals and the age-range in the DCM or AF unaffected control group for genetic association testing. Young unaffected individuals retain the potential to develop DCM or AF in the future; thus including these individuals in the unaffected group would have resulted in misleading genetic association test results. Ideally, only very aged unaffected individuals would have been included in the unaffected group as the certainty that they have not developed DCM or AF would have been high. Although this would have reduced the risk of including an individual in the wrong group, it would have reduced the sample size of the unaffected group to below what is required for genetic association testing. As a compromise the age at which 80% of ultimately affected individuals were affected was determined and this age restriction was utilised to determine at what age to allow an unaffected individual to be included in the unaffected group. In the present study, the age of disease diagnosis was established as significantly different between males and females. Males were diagnosed with DCM/AF on average younger than females, although individuals of both sexes were diagnosed from the age of 1.5 years (Figure 2); this corroborates the findings of Brownlie and Cobb [32]. The age of 80% of all ultimately AF/DCM affected males was established as 6.5 years. Females, however, needed to reach 8.5 years before 80% of the individuals that were diagnosed with AF or DCM had done so. These age restrictions were used to determine at what age it was practical to assign a currently unaffected individual to the “unaffected control” group. Despite the collection of 379 samples across four years, and the use of unstringent age restrictions, there were only 22 individuals not affected by DCM or AF above these two ages. Some of the individuals that were not included in either group had died before reaching the age restrictions, but the majority (261 individuals) either had not reached the age restriction by the end of the study, or had not been heart tested since reaching the age restriction. There was a clear difference in the age of diagnosis between males and females, but the reasons for this are unknown. It is possible that males were heart tested earlier and more regularly than females and thus are overrepresented in earlier diagnoses. The calculations which involved exclusion of individuals diagnosed over the age of 6.5 years of both sexes which had not previously been heart tested did not change the significance threshold of the *t*-test determining differences in the age of diagnosis. This indicated that the differences in the age of diagnosis are likely to be related to the age of disease onset, not age at presentation for heart testing. Strong candidates for further work to investigate these sex differences are hormones which differ in the amount and ratio between the sexes and the different responses to these hormones [43–47]. In light of the differences between males and females, neutering status was of interest. There was not an overall difference in the number of affected cases between neutered and nonneutered IWHs for either males or females; however further investigations into neutering age could be investigated to show whether this affects disease onset or progression.

All loci examined for an association with disease had previously been associated or putatively associated with DCM in IWHs from mainland Europe by Philipp et al. [23]. The current study primarily studied IWHs from the UK and genetic differences between subpopulations of the breed were possible. While this potential genetic difference was possible, it was unlikely as mixing of bloodlines occurs throughout Europe [48]. This was supported by the finding that allele frequencies were broadly similar in both genotyped populations across all loci (Table 5). Given the genetic similarity between the mainland Europe IWH population and the UK IWH population, genetic associations with disease in one population should remain in the other, but this was not the case (Table 6). Only three out of five of the SNPs previously associated with IWH DCM in mainland European dogs were associated with DCM/AF in the current study, and of these only one had the same allele associated with disease (Table 6) [23]. There are a number of possible explanations for this apparent inconsistency between studies. The first is that despite genetic admixture occurring and the similarity in allele frequencies between populations, there was unidentified population substructure. Although this is possible, it is unlikely given the known mixing of bloodlines and the meticulous record keeping required for prestigious pedigree breeds such as IWH [48]. More likely explanations for the discrepancy between populations are the sample size and age at which unaffected individuals were included in the unaffected group. Although our study obtained the largest number of samples in the breed ever collected thus far and the previous study by Philipp et al. [23] was large in comparison to many canine genetic studies, for accurate genotype-phenotype associations to be made a larger number of samples are required. Typical human genotype association studies use over 2,000 individuals, with some using more than 10,000 individuals [49–52]. Philipp et al. [23] used a total of 190 individuals, and in the present study 379 DNA samples were collected but following more stringent inclusion criteria 95 individuals could be included in the genetic association analysis. The age an individual can be considered healthy for DCM genetic association purposes varies between breeds, but it has not previously been based on clear justification [23, 28, 39]. The analysis in the present study shows the age that an unaffected male can be included in the healthy group was younger than an unaffected female (Figure 2). The age that an unaffected female can be included in the unaffected group was 8.5 years; the caveat is that, despite this age restriction, 20% of females that developed DCM or AF did so after this age. The study by Philipp et al. [23] included unaffected individuals of both sexes over the age of 7 years in the healthy group. In the context of the present results this was adequate for males, for which 80% of affected individuals were diagnosed before the age of 6.5 years. In females, however, over 40% of individuals that developed disease in the current study did so after 7 years old (Figure 2); thus the inclusion of females under the age of 8.5 years in the unaffected group is likely to lead to spurious genetic associations in the study by Philipp et al. [23]. In addition the authors did not state whether individuals with AF were excluded from the unaffected group. Although the current study only had 23 individuals in the unaffected group, the genetic associations made are more likely to be correct as individuals were less likely to be inappropriately included in the unaffected group based on age or diagnosis of AF.

Individually, three loci were significantly associated with DCM/AF in the current study, but none of the genotypes conferred a significant increase or decrease in risk compared to the overall population risk of disease (Tables 6 and 7). Combining the significant loci into pairs increased the significance of the genetic associations and the risk of disease for the most risky genotype combinations (Tables 8 [Table tab9] [Table tab10]–11). Despite combining genotypes at pairs of loci, none of genotype risks were significantly increased or decreased relative to the population risk. The combination of genotypes from all three significant loci resulted in a significant genetic association (*p* < 0.0001). Furthermore, the risk of disease in individuals which had the most risky genotypes at all three loci (Chr1 CC, Chr21 GA/GG, and Chr37 AA) was significantly increased compared to the population risk (Table 12). This is consistent with our previous work which showed that information from multiple loci combined could be more informative in predicting disease than individual loci and is the first study to show that the multiple allele effect is important in canine heart disease [31].

Genetic associations identify loci and alleles associated with disease, but to be of relevance to clinicians, breeders, and owners, there is a need to define genotype combinations which significantly increase or decrease the risk of developing disease. Individuals with high risk genotypes can be more intensively monitored for the early signs of disease. With heart testing it is possible to detect disease in advance of overt clinical symptoms and it has been shown that some drugs can increase the time to progression to overt disease, thus improving quality of life and longevity [17]. If high risk genotype combinations can be identified, breeders can reduce the risk of individuals developing disease by ensuring that mating will not result in offspring with genotypes with a high risk of developing disease. The methodology in this study has the potential to supplement those currently utilised in establishing genetic risk scores, as it can account for interactions between loci and dominant or recessive alleles [53–56]. The methodology could be applied to a variety of diseases and traits, and it is not restricted to use in canine disease so it could thus be of use in human genetic counselling. It should be noted that breeding programme management for genetic conditions must be managed carefully to ensure that prevalence of other genetic diseases does not inadvertently increase.

There were no genotypes identified which conferred a significant protective effect, but some genotypes had few (<5) individuals genotyped irrespective of disease status (Tables 7 [Table tab8] [Table tab9] [Table tab10] [Table tab11]–12). From so few genotyped individuals it is not realistic to draw definitive conclusions, but within some of these genotypes there were more individuals without disease than with disease. This indicates that these genotypes may confer a protective effect against the development of DCM/AF compared to the overall population risk; a greater number of affected and unaffected individuals would allow this to be tested. The inclusion of additional individuals could also reduce the 95% confidence intervals for the relative risks at other genotypes, potentially allowing genotype combinations with increased, but not high risk to be confirmed. Increasing the number of individuals genotyped may allow for more stringent age restrictions to be used in determining unaffected individuals which would increase the confidence in any associations made. In the current sample the 90% age restriction would be between 8 and 8.5 years for males and 9 years for females. The use of these age restrictions in the current sample would reduce the number of individuals eligible to be included in the group to 13 which would reduce the power of the genetic association tests still further.

The physiological functions of the DCM/AF associated SNPs have not been confirmed, although some lie within or close to genes [23]. Of the three SNPs associated with DCM/AF in this current study only one, the intronic SNP on chromosome 21 lies within a gene,* PDE3B* [23].* PDE3B* has been shown to have a role in cardiac function [57, 58]. Of particular interest is that there has been reported to be a difference in* PDE3B* mRNA expression between males and females [59]. If* PDE3B* is involved in DCM/AF in IWHs, the differential expression between males and females could explain the difference in the age of onset between the sexes. There were not enough unaffected individuals in the current study to test the SNP for DCM/AF-association in each sex separately. With the inclusion of additional affected and unaffected individuals* PDE3B* could be a promising candidate gene for identifying the function of one aspect of the genetic basis of DCM in IWHs. The two other DCM/AF associated SNPs are not within known genes [23]. These SNPs could lie within regulatory regions or be in linkage disequilibrium with regulatory regions or functional genetic variants [60, 61]. Further work is needed to establish the mode of action of these SNPs.

There are likely to be additional unidentified genetic susceptibility loci in IWH DCM/AF as there were affected individuals with genotypes which appeared to confer a lower risk of disease (Table 12). In addition, there may be unidentified protective loci because there were unaffected individuals with genotypes conferring a higher risk of disease, although these individuals could develop disease later in life as the age restrictions for inclusion in the unaffected group were not very stringent. Further work with a larger number of individuals is required to verify the current genetic associations and identify additional loci associated with IWH DCM as in this study 72 cases and 23 controls were used across a longitudinal study, but with a limited number of tested markers and potential confounding effects such as cryptic relatedness, further studies could provide further insights. In addition, it was interesting to note that our marker allele differed for many markers to that shown in a previous study [23]; further examination of these affected areas could elucidate the differences observed.

## 5. Conclusions

In conclusion, AF is related to DCM in the animals within this study and this novel information enabled individuals with both DCM and AF to be included in the affected group for genetic association testing. In addition, male and female IWHs were confirmed to have different ages of disease onset which allowed more accurate allocation of individuals into the unaffected group. This study also confirms our previous work indicating that multiple loci analysis better predicts the incidence of DCM than single loci alone; however, a larger number of affected and unaffected individuals are required to confirm the associations.

## Figures and Tables

**Table 1:** PCR primer information.

SNP	Primer pair	Forward primer sequence	Reverse primer sequence	PCR fragment length
Chr1: rs21953123 C/T	Initial primers	TCCTCTCCAAACATTAGAAAAAGACC	ACTACATATAGTCAGCTTTACAGATTG	311 bp
	Additional primers	TATCGTACTTTATAATGAAAGATTGAATG	AGAATTTAATATCTGAAAATATATCAACAC	417 bp
Chr10: rs22078677 A/C	Initial primers	CCTGGTGTCAGAAACACAAGgCAC	TCAAGTAGCTTAAACTCTCAGGGCC	374 bp
	Initial forward primer, additional reverse primer		TGTGTATTCATCAGCTCTTCCTAGG	161 bp
Chr15: rs22422063 C/A	Initial primers	AATCATTTGACAGTTAGGTTAACTTCC	CACAATTTGCACATTACACATTATTAAC	375 bp
	initial forward primer, additional reverse primer	GCTGGTAAACATTCTTAAATTCAGCG	ATTTGAGTTTTCCCTCACATTATTGAC	443 bp
Chr21: rs22923291 G/A	Initial primers	GAAACTCCAGTCATGTTTAACTATTTG	GAAGAGCAGTAATGACTAATACgCAG	349 bp
	additional forward primer, initial reverse primer	TGTTCATCTCTCACAGGACTTGAG		410 bp
Chr37: rs24025150 G/A	Initial primers	GGAAACTGGTTTCCAATGAAGAcC	ACGTAACAAGTTTGAAGTGTTGCTG	374 bp
	initial forward primer, additional reverse primer		CCTGCCACCTGTGTTACTTTACGC	133 bp

DCM associated SNP locations in genome (from Philipp et al. [23]), PCR primer sequence information, if the primer pair is the initial or new primers, and resulting PCR product sizes. Primer sequence in lower case indicates a base change from the reference sequence to create a restriction digest site.

**Table 2:** PCR annealing temperatures as determined by temperature gradient PCR optimisation for each primer pair.

SNP	Primer pair	Annealing temperature
Chr1: rs21953123 C/T	All primer pairs	52°C
Chr10: rs22078677 A/C	Initial primers	60°C
	Initial forward primer, new reverse primer	52°C
Chr15: rs22422063 C/A	All primer pairs	52°C
Chr21: rs22923291 G/A	Initial primers	58°C
	New forward primer, initial reverse primer	52°C
Chr37: rs24025150 G/A	Initial primers	58°C
	Initial forward primer, new reverse primer	52°C

**Table 3:** Restriction digest information.

SNP	Enzyme	Units of enzyme per reaction	Restriction site	Cuts allele	Primer pair	Digest fragment sizes
Chr1: rs21953123 C/T	HpyCH4II (NEB, USA)	3	ACTGT	C	Original primers	228 & 83
					New primers	260 & 157
Chr10: rs22078677 A/C	BanI (NEB, USA)	6	GgCACC	C	Original primers	19 & 355
					Original forward primer, new reverse primer	19 & 142
Chr15: rs22422063 C/A	NciI (NEB, USA)	6	CCCGG	C	Original primers	196 & 179
					Original forward primer, new reverse primer	227 & 216
Chr21: rs22923291 G/A	Fnu4HI (NEB, USA)	1	GCTGc	G	Original primers	310 & 39
					New forward primer, original reverse primer	384 & 39
Chr37: rs24025150 G/A	HpaII (NEB, USA)	3	cCGG	G	Original primers	28 & 346
					Original forward primer, new reverse primer	28 & 105

Enzymes, restriction sites, primer pairs, and digest fragment sizes for each SNP.

**Table 4:** The relationship of the risk of disease with the penetrance parameter.

Model	Penetrance		
	a/a	a/A	A/A
Multiplicative	*f*_0_	*f*_0_ *γ*	*f*_0_ *γ* ^2^
Additive	*f*_0_	*f*_0_ *γ*	2*f*_0_ *γ*
Recessive	*f*_0_	*f*_0_	*f*_0_ *γ*
Dominant	*f*_0_	*f*_0_ *γ*	*f*_0_ *γ*

Risk of disease (*f*_0_) of the least risky homozygote (a/a) is related to the genetic penetrance parameter (*γ*) for each additional genotype under multiplicative, additive, recessive, and dominant models. For the multiplicative, additive, and dominant models *γ* is the relative risk of the heterozygote genotype compared to the homozygote genotype with the lowest risk; for the recessive model it is the relative risk of the most at risk homozygote compared to the least at risk homozygote.

**Table 5:** Allele frequencies at each SNP in the data published in Philipp et al. [23] and the current study.

Allele	Published allele frequency	Current study allele frequency
Chr1 C	0.88	0.82
Chr1 T	0.12	0.18

Chr10 A	0.94	0.94
Chr10 C	0.06	0.06

Chr15 C	0.74	0.63
Chr15 A	0.26	0.37

Chr21 G	0.71	0.58
Chr21 A	0.29	0.42

Chr37 G	0.57	0.59
Chr37 A	0.43	0.41

Chr1 refers to the SNP on chromosome 1, Chr10 the SNP on chromosome 10, and so forth, as identified in Philipp et al. [23] and Table 1.

**Table 6:** Mode of penetrance for individual SNPs in relation to IWH DCM/AF.

	Chr1	Chr10	Chr15	Chr21	Chr37
Mode of penetrance	Multigenic	Multigenic	A dominant	G dominant	A recessive
Type of test	Allelic	Allelic	A dominant	G dominant	A recessive
Chi-squared test result	10.61	5.07	1.52	7.67	11.12
df	1	1	1	1	1
Bonferroni corrected *p*	0.0056	0.12	1.00	0.028	0.0043
Published DCM allele	C	A	C	A	G
Current DCM/AF allele	C	C	A	G	A

Mode of penetrance, subsequent genetic association test used, chi-squared test result, degrees of freedom of the chi-squared test, and Bonferroni corrected *p* value for each locus. Also shown are the alleles associated with IWH DCM in Philipp et al. [23] and IWH DCM/AF in the current study. Chr1 refers to the SNP on chromosome 1, Chr10 the SNP on chromosome 10, and so forth, as identified in Philipp et al. [23] and Table 1.

**Table 7:** Risk of developing DCM/AF for each genotype at loci identified as significantly associated with DCM/AF.

Genotype	Risk	Total genotype	Risk relative to population risk	95% CI
Chr1 CC	0.81	54	1.08	0.91 to 1.28
Chr1 CT	0.68	22	na	na
Chr1 TT	0.5	4	na	na
Chr1 CT/TT	0.65	26	0.86	0.64 to 1.17

Chr21 AA	0.61	18	0.81	0.55 to 1.19
Chr21 GA	0.77	39	na	na
Chr21 GG	0.82	33	na	na
Chr21 GA/GG	0.79	72	1.04	0.89 to 1.23

Chr37 AA	0.85	27	1.12	0.93 to 1.36
Chr37 GA	0.70	23	na	na
Chr37 GG	0.73	44	na	na
Chr37 GA/GG	0.72	67	0.95	0.78 to 1.14

The risk of developing disease for each genotype, the total number of individuals with each genotype, the relative risk of each genotype compared to the population risk, and associated 95% confidence intervals. Where dominant or recessive modes of penetrance were established or less than 5 total individuals had a genotype, genotypes were pooled and these are also presented. Chr1 refers to the SNP on chromosome 1, Chr21 the SNP on chromosome 21, and Chr37 the SNP on chromosome 37 as identified in Philipp et al. [23] and Table 1.

**Table 8 tab8:** Chi-squared test results for the genotype associations with DCM/AF for pairs of loci.

	Chr1+21	Chr1+37	Chr21+37
Chi-squared test result	14.16	15.56	24.55
df	3	3	3
Bonferroni corrected *p*	0.0081	0.0042	0.000058

Chi-squared test results, degrees of freedom (df) of the chi-squared tests, and Bonferroni corrected *p* values for genotype associations. Chr1 refers to the SNP on chromosome 1, Chr21 the SNP on chromosome 21, and Chr37 the SNP on chromosome 37 as identified in Philipp et al. [23] and Table 1.

**Table 9 tab9:** The risk of developing disease for each genotype combination at chromosomes 1 and 21 loci.

Chr1	Chr21	DCM/AF risk	Total genotyped	Risk relative to population risk	95% CI
**CC**	**GG/GA**	0.81	48	1.07	0.90 to 1.28
**CC**	AA	0.83	6	1.10	0.76 to 1.60
CT/TT	**GG/GA**	0.74	19	0.97	0.73 to 1.30
CT/TT	AA	0.33	6	0.44	0.14 to 1.37

Total number of individuals with each genotype combination, the relative risk of each genotype compared to the population risk, and associated 95% confidence intervals. The individual genotypes are in bold type if they were identified as conferring increased risk of disease and normal type if they were identified as conferring decreased risk of disease.

**Table 10 tab10:** The risk of developing disease for each genotype combination at chromosomes 1 and 37 loci.

Chr1	Chr37	DCM/AF risk	Total genotyped	Risk relative to population risk	95% CI
**CC**	GG/GA	0.79	38	1.04	0.85 to 1.27
**CC**	**AA**	0.88	16	1.15	0.93 to 1.43
CT/TT	GG/GA	0.61	18	0.81	0.55 to 1.19
CT/TT	**AA**	0.75	8	0.99	0.65 to 1.50

Total number of individuals with each genotype combination, the relative risk of each genotype compared to the population risk, and associated 95% confidence intervals. The individual genotypes are in bold type if they were identified as conferring increased risk of disease and normal type if they were identified as conferring decreased risk of disease.

**Table 11 tab11:** The risk of developing disease for each genotype combination at chromosomes 21 and 37 loci.

Chr21	Chr37	DCM/AF risk	Total genotyped	Risk relative to population risk	95% CI
AA	GG/GA	0.62	13	0.81	0.52 to 1.27
AA	**AA**	0.60	5	0.79	0.38 to 1.63
**GA/GG**	GG/GA	0.75	52	0.99	0.82 to 1.20
**GA/GG**	**AA**	0.89	19	1.18	0.97 to 1.43

Total number of individuals with each genotype combination, the relative risk of each genotype compared to the population risk, and associated 95% confidence intervals. The individual genotypes are in bold type if they were identified as conferring increased risk of disease and normal type if they were identified as conferring decreased risk of disease.

**Table 12:** The risk of developing disease for each genotype combination at chromosomes 1, 21, and 37 loci.

Chr1	Chr21	Chr37	DCM/AF risk	Total genotyped	Risk relative to population risk	95% CI
TT/CT	AA	**AA**	0.50	2	0.66	0.16 to 2.65
TT/CT	AA	GG/GA	0.25	4	0.33	0.06 to 1.81
TT/CT	**GA/GG**	**AA**	0.80	5	1.06	0.67 to 1.66
TT/CT	**GA/GG**	GG/GA	0.73	15	0.97	0.70 to 1.34
**CC**	AA	**AA**	0.50	2	0.66	0.16 to 2.65
**CC**	AA	GG/GA	1.00	4	1.32	1.18 to 1.48
**CC**	**GA/GG**	**AA**	0.93	14	1.23	1.02 to 1.47
**CC**	**GA/GG**	GG/GA	0.76	34	1.01	0.81 to 1.26

Total number of individuals with each genotype combination, the relative risk of each genotype compared to the population risk, and associated 95% confidence intervals. The individual genotypes are in bold type if they were identified as conferring increased risk of disease and normal type if they were identified as conferring decreased risk of disease.
